# Understanding the underlying mechanisms governing spindle orientation: How far are we from there?

**DOI:** 10.1111/jcmm.17526

**Published:** 2022-08-27

**Authors:** Tao Zhong, Xiaoxiao Gongye, Minglei Wang, Jinming Yu

**Affiliations:** ^1^ Medical Integration and Practice Center, Cheeloo College of Medicine Shandong University Jinan China; ^2^ Shandong Cancer Hospital and Institute Shandong First Medical University, Shandong Academy of Medical Sciences Jinan China

**Keywords:** spindle, spindle orientation, spindle orientation model

## Abstract

Proper spindle orientation is essential for cell fate determination and tissue morphogenesis. Recently, accumulating studies have elucidated several factors that regulate spindle orientation, including geometric, internal and external cues. Abnormality in these factors generally leads to defects in the physiological functions of various organs and the development of severe diseases. Herein, we first review models that are commonly used for studying spindle orientation. We then review a conservative heterotrimeric complex critically involved in spindle orientation regulation in different models. Finally, we summarize some cues that affect spindle orientation and explore whether we can establish a model that precisely elucidates the effects of spindle orientation without interfusing other spindle functions. We aim to summarize current models used in spindle orientation studies and discuss whether we can build a model that disturbs spindle orientation alone. This can substantially improve our understanding of how spindle orientation is regulated and provide insights to investigate this complex event.

## 1 INTRODUCTION TO SPINDLE ORIENTATION

The development of multicellular organisms begins post‐fertilization, when rapid cell division occurs in the zygote. The division process is associated with the emergence of diverse cellular functions and assembly of three‐dimensional tissue structures, which rely partially on the orientation of spindle fibres.^1^, ^2^ The directionality of appropriate cell division is established by the spindle orientation, which affects the precise tissue architecture of an organism.^3^, ^4^, ^5^ Spindle misorientation results in various diseases including lissencephaly,^6^ Huntington's disease^7^ and some cancers.^8^, ^9^ hence, the study of spindle orientation will aid in understanding the connection between organismal development and human diseases.

Microtubule remodelling occurs during the formation of the specialized bipolar structure of the spindle fibres. Chromosomes are attached to the spindle microtubules at the kinetochore, which appears as a bridge between the poles. The astral microtubules interact with cortical proteins linking the spindle poles to the cell cortex. Despite research progress in regulating spindle orientation in the past decades, the lack of a suitable universal model has been a key limitation in the study of spindle orientation, suggesting a need to develop an appropriate model. This review presents several causes of spindle misorientation and discusses the possible solutions.

## 2 MODELS FOR STUDYING SPINDLE ORIENTATION

**FIGURE 1:**
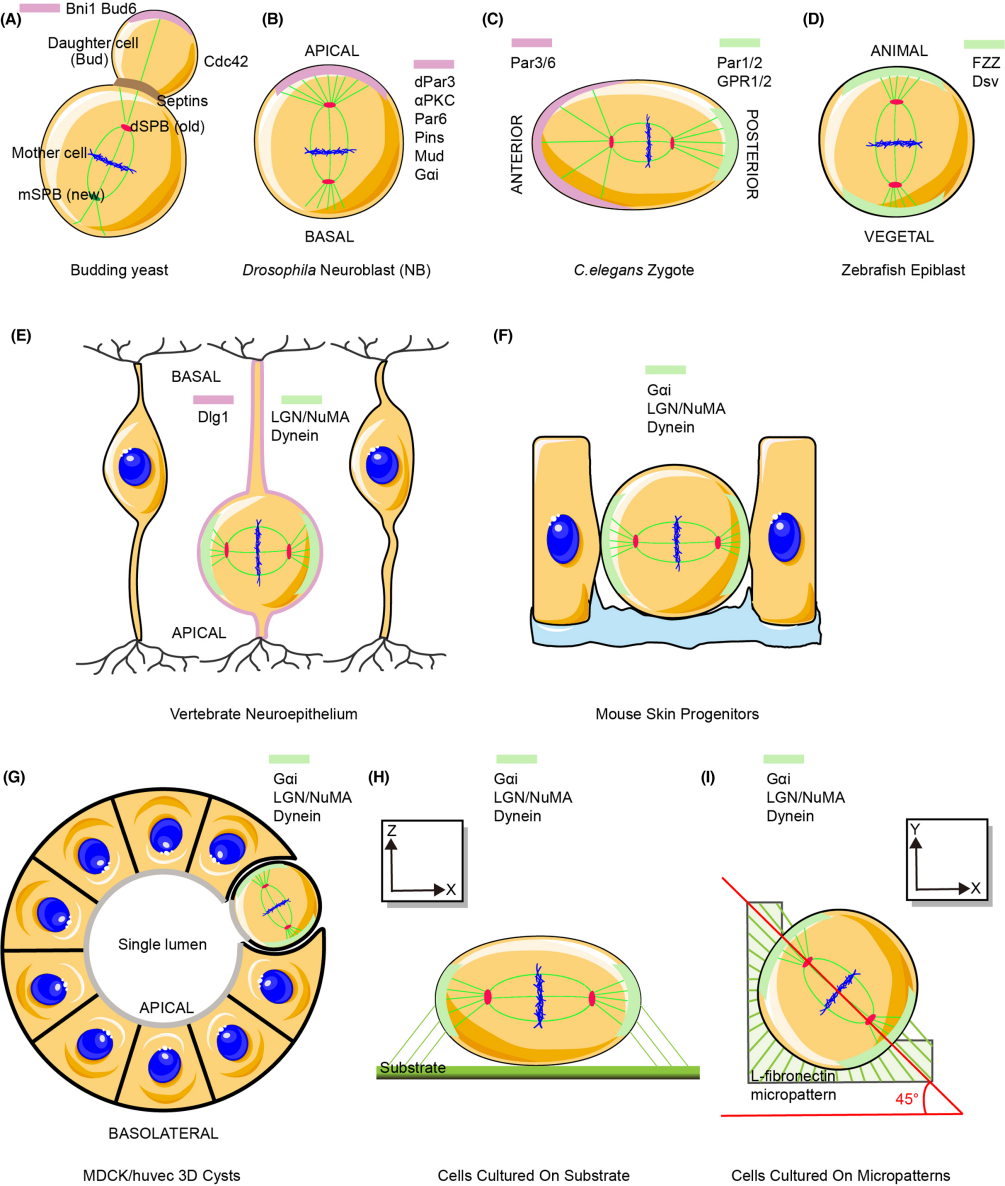
Frequently used models for studying spindle orientation in vivo and in vitro. In vivo models to evaluate spindle orientation in budding yeast (A), *Drosophila* neuroblasts (B), *C. elegans* zygote (C), zebrafish epiblasts (D), vertebrate neuroepithelium (E) and mouse skin progenitors (F). In vitro spindle orientation models of cultured MDCK/HUVECs in Matrigel for real‐time observation of intracellular changes using microscopy (G), cells on fibronectin substrate (H) or micropatterns (I). Conserved polarized factors with different names of homologues in model organisms. Light pink or green represents relevant polar factors in different models. Their asymmetrical spatial distribution at the cell pole will generate two cells with different fates

A correlation between the orientation of the division axis and cell fate has been discovered in *Drosophila* and *Caenorhabditis elegans* ^10^, ^11^, ^12^ and has subsequently been studied in different species.^13^ First, budding yeast was used as a simple system to study asymmetrical spindle polarity^14^ (Figure 1A). In budding yeast, asymmetric targeting of spindle poles to the mother and bud cell compartments orients the mitotic spindle along the mother‐bud axis. This is due to intrinsic functional asymmetry, which generates two cells with different fates. Second, the asymmetric zygotic division and differentiation during early embryogenesis have been investigated in the nervous system of flies^15^ (Figure 1B). Third, mouse skin progenitors, mouse and chick neuroepithelial cells, and fish epiblast cells have been employed to explore the proliferation and differentiation of epithelial cells^16^, ^17^, ^18^ (Figure 1C–F). These in vivo models are important in assessing new regulators and cellular processes associated with development.

However, in vitro models have more advantages in observing intracellular changes using microscopic analysis. The frequently used in vitro models include cells cultured on fibronectin‐coated plates on micropatterns, or using three‐dimensional (3D) culture methods (Figure 1H,I). Among these, cells cultured using 3D systems have been used to study epithelial morphogenesis and lumen formation (Figure 1G). For example, cell polarity with spindle orientation has been evaluated using Madin–Darby canine kidney cells^19^ or human umbilical vein endothelial cells grown in Matrigel.^20^ Defects in spindle orientation lead to the formation of cysts with multiple lumina and inhibit angiogenesis.^21^, ^22^, ^23^ Furthermore, using cells cultured on fibronectin or micropatterns, changes in spindle orientation have been identified by assessing the distribution of actin retraction fibres and astral microtubules.^24^, ^25^ These findings are critical for the diagnosis and treatment of relative diseases.

## 3 CONSERVATIVE HETEROTRIMERIC COMPLEX, GɑI‐LGN‐NUMA, CONTROLS SPINDLE ORIENTATION IN DIFFERENT MODELS

**FIGURE 2:**
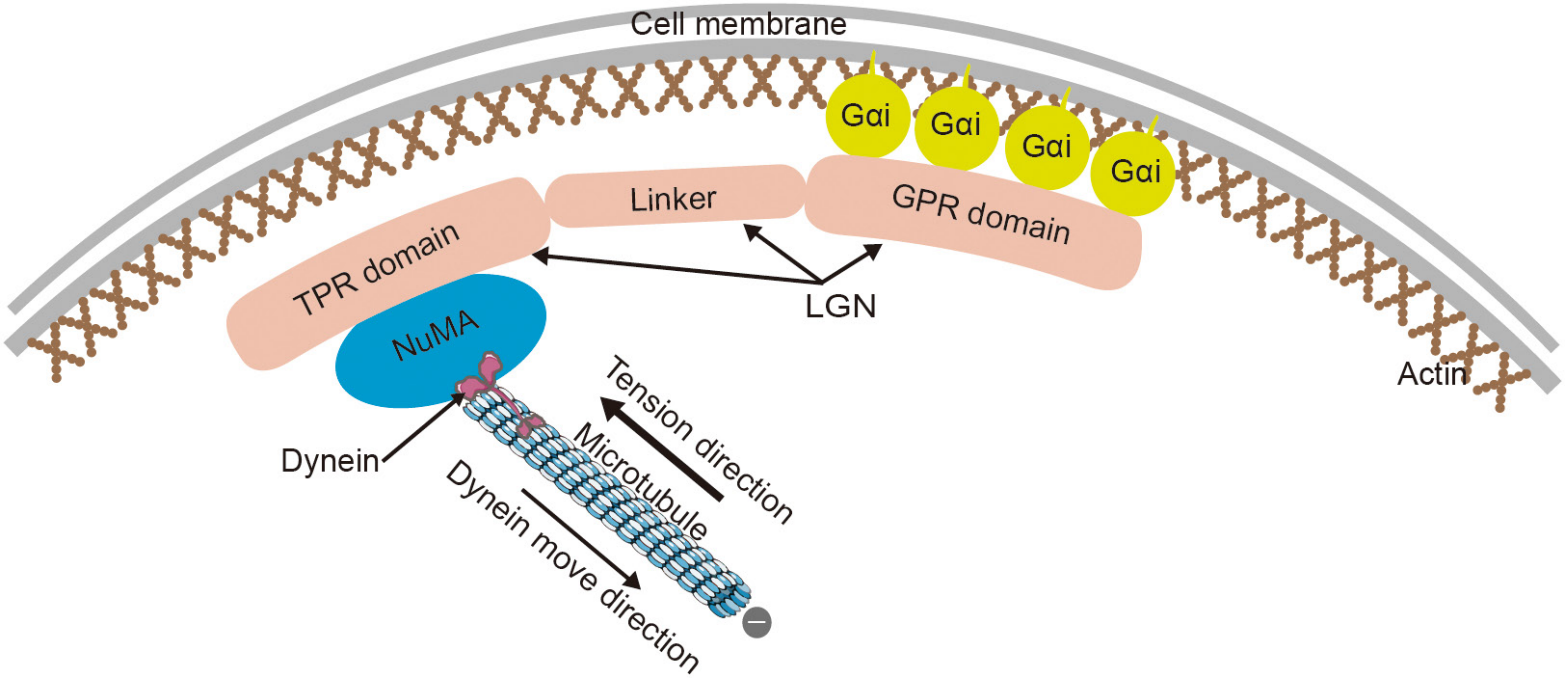
Conservative heterotrimeric complex, Gɑi‐LGN‐NuMA, in spindle orientation controlling mechanism. Gɑi is anchored to the membrane at one end and interacts with the GPR domain of LGN. The TPR domain of LGN mediates the interactions with multiple binding proteins such as NuMA. Dynein directly interacts with NuMA and moves along with the astral microtubule towards the minus end. Therefore, an appropriate pulling force is generated in the opposite direction, which is necessary for proper spindle orientation

A conserved heterotrimeric complex, Gɑi‐LGN‐NuMA, is reportedly involved in regulating spindle orientation both in vivo and in vitro.^26^, ^27^, ^28^ In this complex, LGN is an adaptor molecule of Gɑi. It consists of three main domains: N‐terminal TPR domain, central ‘linker’ domain and C‐terminal GPR domain.^29^ During mitosis, Gɑi is anchored to the membrane by its membrane‐anchored subunits and interacts with the GPR domain of LGN. N‐terminal TPR domain mediates interactions with multiple binding proteins such as NuMA. A functionally unknown ‘linker’ domain connects the two parts together.^30^ Consequently, this complex can locate a specific region of the subcortical domain and recruit the minus‐end‐directed microtubule motor protein, dynein, directly. Dynein movement along the astral microtubule can generate a pulling force on the spindle pole, orienting the spindle at an appropriate plane and position^31^ (Figure 2).

Besides, the coiled‐coil domain of NuMA has been verified as a hairpin that can interact with LGN, dynein and microtubules simultaneously.^32^ Cortical localized NuMA is also frequently observed in dividing cells,^33^ which require NuMA phosphorylation.^34^ Similarly, Gɑi subunits localizing to the plasma membrane are myristoylated. The modified Gɑi protein attaches to the cortex providing an anchor to the TPR domain of the LGN complex.^35^ Remarkably, this conserved complex is known as Gɑi‐Pins‐Mud in *Drosophila* and GOA1/GPA16‐GPR1/2‐LIN5 in *C. elegans* (Table 1).

**TABLE 1:** Genes mentioned in this review and their homologues in different model organisms

*C. elegans* 85, 86	*Drosophila* ^87^	Vertebrates^13^
*GOA1*/*GPA16*	*Gɑi*	*Gɑi1*, *Gɑi2*, *Gɑi3*
*GPR1/2*	*Pins* (partner of inscuteable, Rapsynoid)	*LGN* (*GPSM2*, *mPins*)
*Lin‐5*	*Mud*	*NuMA* ^88^
–	inscuteable	*Insc* (*mInsc*)
*Par3* ^69^	*Bazooka*	*Par3*
*DLG‐1*	*Dlg* ^17^	Dlg1

## 4 FACTORS REGULATING SPINDLE ORIENTATION

Specialized bipolar structures of spindle fibres are affected by several factors. They can be roughly classified as internal, external and geometric cues. They have a considerable influence on the spindle orientation. However, they eventually affect spindle orientation machinery or cell cortex interactions with astral microtubules. Here, we discuss the different factors in detail.

### 4.1 Internal cues

The assembly of the spindle orientation machinery in the cell requires an intact actin cortex and normal astral microtubules. The cortex can effectively generate a stable force to organize the spindle at appropriate angles.^31^ Latrunculin A or cytochalasin D treatments lead to spindle orientation defects, affecting cell fate.^36^ Additionally, remodelling the stiff actin during mitosis can provide sufficient force to pull the spindle orientation machinery.^37^, ^38^ However, the precise location where this force is generated is not known. Furthermore, abnormal astral microtubules perturb the spindle dynamics and stability, or interaction with cortical proteins, leading to misorientation, irrespective of their defective nucleation/anchoring.^39^, ^40^, ^41^ In addition, some proteins and kinases in the cell can contribute to the formation or stabilization of microtubules, thereby affecting spindle assembly or functions. For example, cylindromatosis is a deubiquitinating enzyme, which directly binds to the microtubules and regulates astral microtubule stability and dynamics via lysine 63‐linked ubiquitin hydrolysis.^42^ Polycomb repressive complex 1, a minus‐end kinesin protein of the microtubule, is also important for the proper assembly, dynamics and positioning of the mitotic spindle.^43^, ^44^

### 4.2 External cues

Extracellular signals from the cell surface can control spindle orientation. The extracellular matrix (ECM), a structure composed of proteins and polysaccharides secreted by cells, is located on the cell surface or between cells and affects spindle orientation directly or indirectly.^45^, ^46^ One of its components, β‐integrin, can interact with focal adhesion kinase and talin to regulate spindle alignment.^47^, ^48^ Besides, β‐integrin knockout mice display random spindle orientation during skin stratification^49^ and luminal formation.^50^ β‐Integrin has been proved to be a key element in establishing apical‐basal polarity for spindle orientation and the relative molecular location of LGN, NuMA and ɑPKC.^27^ Besides β‐integrin, other ECM components such as exopolysaccharides are involved in spindle orientation through sulfation and uronic acid epimerization.^51^ During mitosis, JAM‐A control spindle orientation through Cdc42, further regulating cortical dynein localization.^52^ Caveolin‐1 can translate the interphase adhesion geometry to mitotic spindle orientation in a RhoA‐dependent manner.^53^ During kidney morphogenesis and repair, renal tubular epithelial cells lacking the transmembrane receptor Plexin‐B2 or its semaphorin ligands fail to correctly orient the mitotic spindle, leading to severe defects in epithelial architecture and function.^54^ Interestingly, β‐integrin, a transmembrane protein, can interact with the ECM and cortical molecules. Therefore, we speculate that β‐integrin may act by transmitting messages from the ECM to the intracellular cortex, possibly regulating spindle orientation through microtubule‐associated proteins or actin cytoskeleton interaction. Future studies are necessary to verify the difference in the role of β‐integrin between normal cells and spindle misoriented cells.

### 4.3 Geometric and external force cues

In the past, cells were considered to divide along their longest axis. This is called the ‘Hertwig rule’. It has been indicated that the spindle in a mitotic cell can perceive cell shape changes to realign itself along the longest axis.^55^ Continuous remodelling allows sufficient space for the formation of the spindle. In cells cultured on fibronectin or micropatterns, the distribution of actin retraction fibres dictates the orientation of the spindle.^22^ Myosin 10 is considered the linker between actin and microtubules in this context.^56^ Considering the relationship between myosin and dynein proteins, the formation of the classical structure of the LGN/dynein complexes is a conservative mechanism of spindle orientation.^57^ Artificial altering of cell shapes causes chromosome missegregation.^58^ Moreover, changes in the spindle angles have been confirmed under external magnetic field actions,^59^ indicating that magnetic force can serve as a kind of external force to regulate spindle orientation. However, a recent study revealed that cell division orientation in vivo is not determined by cell shape but rather by local anisotropies in cell mechanics.^13^, ^60^ Studies have shown that the development of *Drosophila* wing is not dependent on its shape.^61^ Furthermore, tissue tension and non‐interphase cell shape determine cell division, as confirmed in *Drosophila* follicular epithelium.^62^ This tissue tension at compartmental boundaries is actomyosin‐driven tension.^63^, ^64^ In summary, more external forces affect spindle orientation, which need further evaluation.

## 5 CONCLUSIONS AND PERSPECTIVE

The findings on the molecular mechanisms that control the orientation of the mitotic spindle reveal that the conservative heterotrimeric complex Gɑi‐LGN‐NuMA regulates spindle orientation in different species. However, the molecular interplay to regulate the recruitment and maintenance of LGN to the cellular cortex is still unknown. The knockdown of LGN or NuMA results only in weak spindle orientation phenotypes,^65^, ^66^ suggesting an involvement of additional pathways. Additionally, different mechanisms in different tissues contribute to the process of spindle orientation regulation, highlighting the need to establish a universal spindle orientation model to study related issues.

A specialized bipolar spindle orientation machinery plays an irreplaceable role in regulating spindle angles.^67^ It relies on the interaction of astral microtubules with cortical proteins to dictate spindle position and orientation.^68^, ^69^ Therefore, factors concerning spindle morphology and/or behaviour are likely to affect their orientation.^70^, ^71^, ^72^ Moreover, cortical determinants and astral microtubules are not passive participants during this process. They are the core structural components of the spindle orientation machinery. The cortical proteins tune external force transmission into cells to finely regulate the spindle orientation. Understanding cellular sensing for ‘external pressure signal’ and its transmittance into cells is essential. The transmembrane protein β‐integrin is worth investigating to discover a more precise mechanism for regulating spindle orientation and establishing an extracellular controllability model. In addition, most relative proteins interacting with microtubules, especially astral microtubules, have internal cues involved in spindle orientation regulation. Indeed, several proteins perturb spindle orientation by affecting astral microtubules. For example, microtubule plus‐end protein EB1 can stabilize astral microtubules to regulate spindle orientation through phosphorylation.^73^ Human microcephaly ASPM protein is a spindle pole‐focusing factor that regulates orientation by affecting the dynamics of astral microtubules.^74^ Studying the differences between astral and other microtubules to control microtubule behaviour may be a new method of establishing a spindle orientation model. Meanwhile, protein kinases have been proposed to influence spindle orientation.^75^

Aurora‐A kinase can regulate αPKC/Numb cortical polarity and spindle orientation to inhibit neuroblast self‐renewal in *Drosophila*.^76^ In fission yeast, mitogen‐activated protein kinase ensures proper mitotic spindle orientation via the actin checkpoint.^77^ αPKC‐mediated phosphorylation of apical Pins controls epithelial spindle orientation.^78^

Adenosine‐5′‐monophosphate‐activated protein kinase has been found to regulate mitotic spindle orientation through the phosphorylation of the myosin regulatory light chain.^79^ However, kinases contribute to cell signalling and complex life activities. Therefore, setting up a new spindle orientation model affected by kinases and their associated pathway will be useful.

Previous studies have compared cell division in vitro and tissue development in vivo under controlled spindle orientation.^80^, ^81^, ^82^, ^83^, ^84^ The role of spindle orientation in normal and pathological development and homeostasis has been acknowledged. However, due to the lack of a suitable universal model, differential findings among the models cannot be confirmed. As spindle orientation is poorly understood, future work should aim at summarizing the similarities and differences. Overall, developing a universal spindle orientation model is necessary to study diseases and suggest possible treatments for pathologies caused by spindle misorientation.

## AUTHOR CONTRIBUTIONS

**Tao Zhong:** Software (equal); writing – original draft (lead); writing – review and editing (lead). **Xiaoxiao Gongye:** Writing – original draft (equal). **Minglei Wang:** Software (equal); writing – original draft (equal). **Jinming Yu:** Conceptualization (lead); funding acquisition (supporting); project administration (lead); writing – review and editing (lead).

## CONFLICT OF INTEREST

The authors confirm that there are no conflicts of interest.

## Data Availability

Data sharing is not applicable as no new data were generated.
